# Accountable priority setting for trust in health systems - the need for research into a new approach for strengthening sustainable health action in developing countries

**DOI:** 10.1186/1478-4505-7-23

**Published:** 2009-10-24

**Authors:** Jens Byskov, Paul Bloch, Astrid Blystad, Anna-Karin Hurtig, Knut Fylkesnes, Peter Kamuzora, Yeri Kombe, Gunnar Kvåle, Bruno Marchal, Douglas K Martin, Charles Michelo, Benedict Ndawi, Thabale J Ngulube, Isaac Nyamongo, Øystein E Olsen, Washington Onyango-Ouma, Ingvild F Sandøy, Elizabeth H Shayo, Gavin Silwamba, Nils Gunnar Songstad, Mary Tuba

**Affiliations:** 1 DBL - Centre for Health Research and Development, Faculty of Life Sciences, University of Copenhagen, Thorvaldsensvej 57, DK 1871 Frederiksberg, Denmark; 2 Department of Public Health and Primary Health Care (ISF) and Centre for International Health (CIH), University of Bergen, PO Box 7804, 5020 Bergen, Norway; 3 Umea International School of Public Health (UISPH) Umea University, SE 90185 Umea, Sweden; 4 Centre for International Health (CIH), University of Bergen, PO Box 7804, 5020 Bergen, Norway; 5 Institute of Development Studies, University of Dar Es Salaam, PO Box 35169, Dar Es Salaam, Tanzania; 6 Centre for Public Health Research, Kenya Medical Research Institute (KEMRI), Box 20752, Nairobi 00202, Kenya; 7 Department of Public Health, Prince Leopold Institute of Tropical Medicine, Nationalestraat 155, B 2000 Antwerpen, Belgium; 8 Department of Health Policy, Management and Evaluation and the Joint Centre of Bioethics, University of Toronto, 88 College St, Toronto ON, M5G-1L4, Canada; 9 Department of Community Medicine (DCM), School of Medicine, PO Box 50110, University of Zambia, Zambia; 10 Primary Health Care Institute (PHCI) PO Box 235, Iringa, Tanzania; 11 Institute of Economic and Social Research (INESOR), PO Box 30900, University of Zambia, Zambia; 12 Institute of Anthropology, Gender and African Studies (IAGAS) University of Nairobi, PO Box 30197, Nairobi 00100, Kenya; 13 Haydom Lutheran Hospital, PO Mbulu, Manyara, Tanzania; 14 National Institute of Medical Research (NIMR), PO Box 9653, Dar Es Salaam, Tanzania

## Abstract

Despite multiple efforts to strengthen health systems in low and middle income countries, intended sustainable improvements in health outcomes have not been shown. To date most priority setting initiatives in health systems have mainly focused on technical approaches involving information derived from burden of disease statistics, cost effectiveness analysis, and published clinical trials. However, priority setting involves value-laden choices and these technical approaches do not equip decision-makers to address a broader range of relevant values - such as trust, equity, accountability and fairness - that are of concern to other partners and, not least, the populations concerned. A new focus for priority setting is needed.

Accountability for Reasonableness (AFR) is an explicit ethical framework for legitimate and fair priority setting that provides guidance for decision-makers who must identify and consider the full range of relevant values. AFR consists of four conditions: i) relevance to the local setting, decided by agreed criteria; ii) publicizing priority-setting decisions and the reasons behind them; iii) the establishment of revisions/appeal mechanisms for challenging and revising decisions; iv) the provision of leadership to ensure that the first three conditions are met.

REACT - "REsponse to ACcountable priority setting for Trust in health systems" is an EU-funded five-year intervention study started in 2006, which is testing the application and effects of the AFR approach in one district each in Kenya, Tanzania and Zambia. The objectives of REACT are to describe and evaluate district-level priority setting, to develop and implement improvement strategies guided by AFR and to measure their effect on quality, equity and trust indicators. Effects are monitored within selected disease and programme interventions and services and within human resources and health systems management. Qualitative and quantitative methods are being applied in an action research framework to examine the potential of AFR to support sustainable improvements to health systems performance.

This paper reports on the project design and progress and argues that there is a high need for research into legitimate and fair priority setting to improve the knowledge base for achieving sustainable improvements in health outcomes.

## Introduction

Efforts to strengthen district level planning in poorer countries using technical approaches based on burden of disease measures, cost effectiveness analysis, and capacity considerations have not achieved intended sustainable improvements [1]. They emphasize a narrow range of values without reaching neither adequate consensus between them nor the desired acceptance and changes at the operational level [2]. Technical approaches that do not permit deliberation about the full range of relevant values tend to produce disagreement and controversy, as eg. between efficiency and equity. Already a decade ago, Holm argued that the time has come to say 'goodbye to the simple solutions' [3] and a number of authors have strongly urged for innovative approaches [4-6]

**Figure 1:**
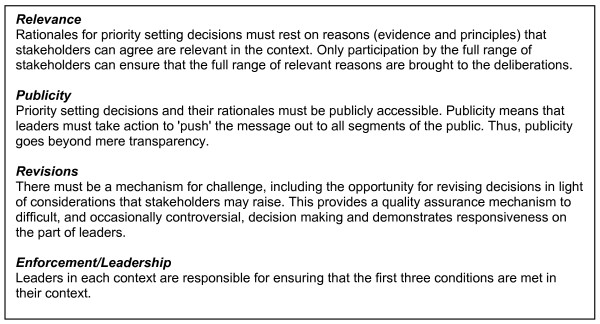
**The conditions of Accountability for Reasonableness**.

The necessity for a new focus on legitimate and fair priority setting has emerged [7,8], including consideration of other relevant values such as trust in health care [9]. Accountability for Reasonableness (AFR) is a framework for legitimate and fair priority setting [10,11]. AFR provides decision makers with an approach to the adjudication of relevant but competing values that are perceived to be legitimate and fair. AFR consists of four conditions: i) relevance to the local setting, decided by agreed criteria; ii) publicizing priority-setting decisions and the reasons behind them; iii) the establishment of revisions/appeal mechanisms for challenging and revising decisions; iv) the provision of leadership to ensure that the first three conditions are met. The four conditions are further described in Figure 1.

AFR thus provides structure to the process of priority setting that helps stakeholders to establish priorities within their specific contexts, while taking into account the available resources and regulatory conditions. The resultant priorities therefore have better chance of gaining acceptance and support, leading to sustainable health action and improved health outcomes [12,13]. AFR based improvement of priority setting can be accommodated in any planning and management procedures from strategic level to facility operations. The focus on the process of priority setting rather than on the priorities as such is an innovation that responds to the long standing calls for increased focus on process and context to enhance the delivery of quality service [6]. AFR provides a framework for such focus, has hence become an important reference for priority setting [14], and has been assessed and in various degrees and forms been incorporated in health services settings in several countries including Canada, United Kingdom, New Zealand, Sweden and the USA as well as in more resource poor settings such as Mexico [15], Zimbabwe [16], and Uganda [13]. Introduction and scientific assessment of AFR is ongoing in most of these and in several other countries, including those addressed in this paper.

**Figure 2:**
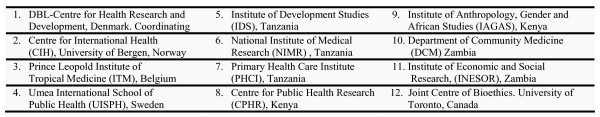
**Participating Institutions**.

The paper describes a five year EU funded research project (Contract PL 517709): *Strengthening fairness and accountability in priority setting for improving equity and access to quality health care at district level in Tanzania, Kenya and Zambia*, which applies and evaluates AFR in a Developing Country context. The research process is indicated by the short title: REsponse to ACcountable priority setting for Trust in health systems (REACT). Participating institutions are presented in Figure 2.

The objectives of the project are to strengthen the legitimacy and fairness of priority setting at district level in Tanzania, Zambia and Kenya, and to evaluate potential changes in quality, equity and trust pertaining to health services and interventions. We are thus applying the AFR conditions in a comprehensive manner and in a broad organisational setting. Additionally we assess its feasibility at district level in low income countries, where improvement of health systems performance within currently available resources is even more challenging. Furthermore, the district level has been purposefully selected for its ideal position of being in direct relation to the national health services as well as providing direct services to the communities. This paper is aimed at providing a description of REACT and how it was designed, developed and is progressing as well as indications of new insight that is expected from future publications of findings. We wish through the REACT project approach and evaluation to provide further insight into potentials for AFR to provide sustainable strengthening of health systems based on well defined processes of empowerment and democratization.

## Main research design

**Figure 3:**
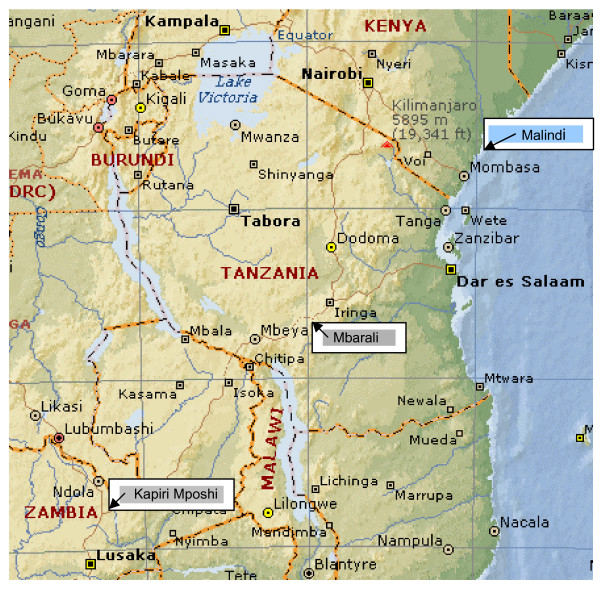
**District locations**.

The REACT project adapts, applies and evaluates priority setting approaches according to the AFR framework in Mbarali District in Tanzania, Malindi District in Kenya and in Kapiri Mposhi District in Zambia (Figure 3).

These districts were chosen to be fairly similar in disease burden as well as to represent common district health systems situations in their countries. Their populations range from 241-342.000 with different density, malaria tops the list for recorded outpatient attendance followed by respiratory tract infections and diarrhoeal diseases. HIV-AIDS is of high concern and service resources are limited but differ in their organisation.

The project applies AFR through a participatory and interdisciplinary action research design. Each study district represents a case, in which decision-making is studied from district to community levels. Each case will be analysed separately as well as in comparison with the other study districts.

A case study is 'an empirical inquiry that investigates a contemporary phenomenon within its real-life context' [17]. Case studies are structured yet flexible approaches that are used to describe institutions and their actions This is the appropriate method because priority setting in healthcare institutions is complex, context-dependent, and involves social processes. Action research is research conducted in partnership with members of the community or setting in question with the specific purpose of bringing about structural or cultural change. It 'involves researchers and non-research partners in joint problem definition, selection of research methods, data collection, analysis, plans and actions. Action research is an excellent way for researchers and local participants to collaborate on developing and implementing a plan to achieve a common goal. It provides rigorous research methods to capture and describe new types of knowledge while making change in an organization [18].

**Figure 4:**
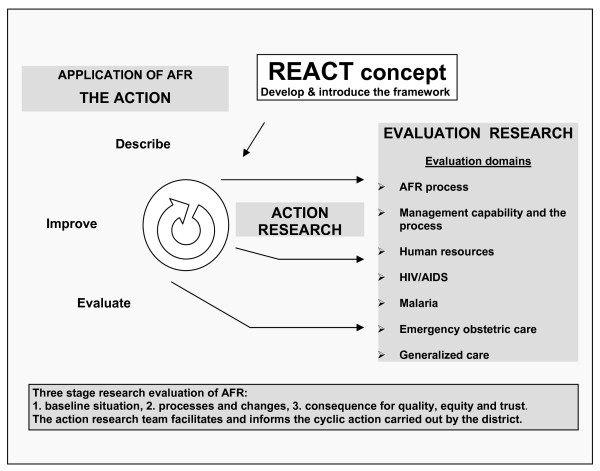
**The REACT conceptual Framework**.

The REACT research process involves an intervention, which is the application of AFR, a scientific assessment of the intervention process as well as an evaluation of the applicability of its conditions to priority setting and the subsequent effects on health systems. The main conceptual framework for REACT is shown in Figure 4.

The application of AFR includes i) describing priority setting in the district, ii) evaluating the description using AFR, and iii) implementing improvement strategies in a continuous process to address gaps in AFR conditions [10].

As an action research project focusing on priority setting, there is an immediate effect on the actual practice. It therefore does not only investigate the application of AFR, but it also examines effects of the application on priority setting within the selected evaluation domains. They include the AFR process itself, management, human resources, HIV/AIDS, malaria, emergency obstetric care and general care. We have defined this range of evaluation domains as representative of important health systems components and services. To measure effects within the evaluation domains we have identified the value areas quality, equity and trust as focuses of analysis. The evaluation is based on qualitative and quantitative methods in a cross disciplinary design. Contextual socio-cultural factors of relevance and other concurrent interventions in the districts studied are also examined in order to improve the understanding and evaluation of the results.

We realize that trust in health systems is influenced by quality and equity but also by a number of other factors and therefore merits its own focus. Trust can be considered as a proxy indicator for legitimacy, accountability and responsiveness. Utilization is associated with trust in health systems. A major focus is thus on trust between users and the health system. Trust in health systems is dependent upon the cultural processes inherent within respective local societies and communities. Trust will also be examined in the relations between levels of the health system, between health providing or supporting organizations and in personal relations within organizations.

The AFR process is carried out by the District Health Management Team (DHMT) with support from an Action Research Team (ART) consisting of a few selected researchers from the research institutions and selected members of the (DHMT) of each study district.

The action research is carried out by the ART with support from the rest of the research team members in the research institutions.

The evaluation research is carried out by the research institutions, but results from the baseline and monitoring that can assist the AFR process in the district are communicated to the DHMT through the ART.

The chosen initial focus for application of AFR are the DHMT's and their main collaborators, aiming to increasingly include health facilities, communities, other sectors and stakeholders. Several papers have shown the applicability of AFR in health care organizations, also in developing countries [16,19-21]. We are taking a similar organizational approach aiming first to consolidate AFR processes within the DHMT, which if successful shall increasingly involve others in the application of AFR in their relations with the DHMT and possibly also in their own organisations and settings. We do not know beforehand how far we shall be able to facilitate application of AFR beyond the DHMT, but we shall consider and active practice of AFR by the DHMT as a first important outcome. However the wider aim of AFR remains to contribute to public empowerment and democratization in all aspects of the involved organisations and the wider society [10] concerned with health.

## Data collection methods

Data and information are collected at the district centre, facility and community levels. They have provided a comprehensive baseline by 2006 for the monitoring of progress and for the final evaluation in 2010. Communities and facilities have been sampled in a three stage stratified sampling approach. Qualitative and quantitative information and data have been collected from the same enumeration areas as defined in the latest country census and from the facilities serving these areas.

Continuous process data are collected for the AFR application and other in depth and associated (such as PhD) studies are carried out within the project period. They include:

i) In-depth interviews (IDI) and focus group discussions (FGD) in relation to the AFR application, the social context and the six evaluation work packages (see fig 4) based on specific guides for different topics and types of respondents,

ii) A population-based questionnaire survey of 2000 persons covering core issues from the evaluation work packages,

iii) documentary review of relevant national and district based documents,

iv) facility inventories and routine data.

v) Other tools are being applied in studies that provide for more in-depth assessment of HIV/AIDS preventive efforts in places where exposures are highest. Similarly, a review of facility records is carried out to assess and document the extent of unmet obstetric needs. These studies meet the opportunity to use methodologies currently being applied by two REACT institutions and align them to the REACT evaluation design.

vi) The AFR processes are being recorded in the form of minutes and observation reports of meetings and activities within as well as outside the district.

Ethical approval has been obtained from relevant authorities in all the three study countries, and in Denmark where the project coordination is located.

## Analytical framework

The AFR conditions and the three core values of quality, equity and trust shall be assessed including their determinants and the relations between them at all levels from users/communities to national. The choice of quantitative and qualitative data, indicators, themes and analytical approaches is based on the extensive formative work that was done at in the beginning of the project through a series of project workshops joining all participating institutions and covering in a main sequence and continuity the development of concepts, tools, data handling processes, analytical approaches, AFR practical application and a number of qualitative and quantitative smaller capacity building and software application workshops.

**Figure 5:**
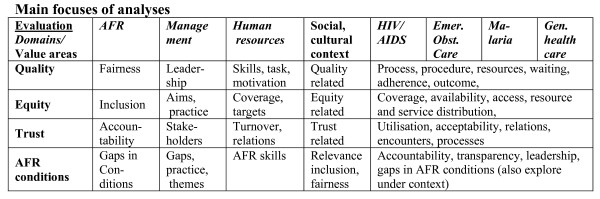
**The analytical framework**.

Figure 5 provides an overview of the evaluation domains and identifies main focuses of analyses for each of them within the selected value areas quality, equity, trust and the AFR conditions. Indicators and analytical themes have been developed within each of the value areas and may be further refined based on insight from the qualitative studies. Results will be interpreted in the context of one of the four value areas based on comparisons in and between countries.

## Implementation of the project

From late 2005 till September 2008, project development has included a sequence of workshops in the study countries and one held in Canada that focused on latest concepts and application of priority setting concepts and practice in mainly developing countries.

Concurrently, baseline data have been collected and the implementation of AFR was started. The AFR conditions and values of accountability and inclusiveness were also applied within the REACT research group and particularly reflected in the standard operational procedures for the project management and the research thus leading to a sense of ownership of the project in each participating institution in both Europe and Africa. The project Steering Committee (SC) includes all team leaders and was established at the start of the first workshop. From the onset it has made all major strategic and design decisions in a participatory manner. However, developing an international, multi-centre, inter-disciplinary, process focused action research study and research partnership has proven to be labour- and communication intensive, leading to some delays. We aim to publish these experiences from the project.

The AFR-approach to priority setting has been introduced in scientific forums across the study countries and to selected country stakeholders in health. Oral and poster presentations of the project have been made in national and international forums. In addition, many other opportunities for introducing and discussing the project at all partner institutions in Europe and Africa have been used. This is in response to the aim of the REACT project to influence health system activities right from the start. Apart from several reports and other briefings, REACT has also been referred to in publications [22,23].

## Reflections

This section picks up on a few issues and concerns that the study is aware of and hopes to clarify based on its results. It is not an attempt at any adequate critical analysis in these areas and as such not based on any adequately referenced explicit review of current evidence. However, it briefly presents and assesses some initial project experiences and considerations related to other current research and development issues. As they stand in the following they rather illustrate some potential areas in which the project hopes to add relevant knowledge.

The AFR action research process has established a close working relationship with the current district health management teams and therefore, from inception, put in place a research-into-action approach. The project elements of accountability, fairness and inclusiveness support good governance and democratization within health systems management, especially as relations and involvement of users increase.

The very resource poor settings targeted by REACT have raised the question whether there will be commitment and motivation for AFR-based continuous priority setting since the intervention does not provide additional resources. Experiences so far strongly indicate that AFR is needed and may be more in demand in such settings, since the priority setting decisions are even more difficult where resources are particularly scarce [13]. Also districts that have elaborated strong plans and realistic budgets based on their own local priorities may be better able to effectively utilize their resources and therefore also to attract additional financial or other resources.

The possibility that each country's Ministries of Health would view AFR as a challenge to the national guidelines was considered during project introduction. This potential contradiction between local priority setting based on AFR and the national priority setting process has attracted lively discussions and shall be examined by the study. However, no concerns in this respect were expressed when the study was introduced at national levels. Even if current macro-level priority setting may limit some opportunities for the application of AFR and subsequent better achievement of sustainable health action, it leaves major spaces for district and other local priority setting that address the numerous local conditions and options that differ from district to district. Legitimate and fair priority setting at the district and local levels is thus a possible and even necessary addition to the national guidance. However, existing guides and procedures are in the study districts viewed as too limiting to a more inclusive and accountable priority setting.

We find it reasonable to assume that improved priority setting will contribute to stronger health systems and improved health outcomes in the study districts. However, changes in service output and outcomes may be due to several concurrent developments and other factors not associated with the AFR conditions and values. We shall therefore through the documentary review and other end of project data gathering refer to such changes and relate them to the AFR conditions and in what way they have been likely to support or constrain the application of AFR. Concurrently with the intervention, other efforts towards more accountability, for instance, may strengthen AFR conditions and political or other conflicts may weaken them.

In this project we focus on the case study processes in each study district and we have not included districts that are not being exposed to the AFR intervention. This means that results shall emerge from situations and developments in each study district and be compared between study districts as well. The difference that the intervention may make shall be analyzed in depth within the broader both district and national context. We have therefore not found a sufficient added advantage of establishing control districts. Such would also have constrained resources for the scope and depth of studies in the intervention districts.

This project does not introduce any supplementary financial, human or physical resources. It was therefore considered whether buy-in by local decision-makers would be reduced. This has been explained by the research nature of this project and its intervention as not being the common type of additional funds driven development project. Once that was realized one high level official in the Ministry of Health in Kenya particularly welcomed the project as not diverting national priorities based on funding conditions. It shall be examined whether the lack of financial input by the project to the district development and a support to district participants only in the form of routine allowances have been constraints to project progress. So far it seems that the empowerment of local actors is in itself a strong motivating factor. This corresponds to other insight from human resources management that there are other strong motivators than increased resource provision and financial gain. We expect to document this argument further e.g. in the human resource evaluation domain of REACT.

As the districts realize that they do not have the capacity to live up to all general service and specific programmatic expectations, the AFR approach can assist in reaching fair and realistic compromises. Such explicit choice may be better than just letting priorities be set on the basis of hidden criteria, resulting in unknown variation from program to program and from district to district. Such situation seems to currently weaken the evidence base for national evaluation and in planning cycles.

## Conclusion

The traditional approach to improving priority setting using technical fixes has not achieved sustainable improvements in health systems or health outcomes because it emphasizes a narrow range of values about which there is no consensus. The REACT approach, using AFR as its conceptual framework, focuses on improving the legitimacy, accountability and fairness of priority setting at the district level, and on generating new knowledge on application of values in priority setting among decision makers such as health service managers, health workers and members of the community.

Attribution of cause and effect represents a challenge in an action research project that focuses on the main outcome indicators areas trust, quality and equity in a district health systems setting that is under multiple influences not controlled by the project. However, all changes shall be assessed through the AFR framework conditions and values and the insight from the application of AFR shall assist in identifying direct AFR related changes and other concurrent changes that may have influenced the outcomes. The development and implementation of an international, multicentre, inter-disciplinary, process focused action research study has proven to be time-consuming, but scientifically enriching. It has improved insight into a complex real life organisational situation. The project is expected to provide a better understanding of local priority-setting processes and how these can be strengthened to contribute to more locally relevant and more sustainable health action.

## Competing interests

The authors declare that they have no competing interests.

## Authors' contributions

JB sent the application for funding to the EU and coordinated the project, its design, data collection, analysis and drafted the paper. PB assisted in coordination and provided adjustments to the paper. AB contributed to qualitative methods development, data analysis approaches and provided major input to the paper. A-KH recommended on the study intervention, its documentation and hosts the REACT master data base. KF was key to design of the quantitative methods and to their analysis and contributed to the write up. PK overall coordinated all work in Tanzania and did some of the qualitative data analysis. YK overall coordinated all work in Kenya. GK overall coordinated all work of the CIH team. BM was the chairman of the Scientific Committee judging all scientific development and drafting a number of standard operating procedures. DKM contributed the main insight on the key concept for the application to EU and advised throughout. CM was part to quantitative data collection surveys and was later delegated the coordination responsibility in Zambia. BN had particular focus on the district process development and support in Tanzania. TJN coordinated the qualitative studies in Zambia. IN coordinated qualitative studies in Kenya. ØEO developed the main project concepts and methods for the application to EU and provided important input to all subsequent work. WO trained in qualitative methods for REACT researchers and coordinated and participated in the field work and analysis in Kenya. IFS had a particular focus on coordinating the specific HIV/AIDS study. EHS had a particular focus on qualitative study data collection and analysis in Tanzania. GS Coordinated the Zambian team from the onset. NGS coordinated the training in qualitative software and ensured qualitative data consistency and completeness. MT had a particular focus on qualitative study data collection and analysis in Zambia. All authors contributed to the design of the overall study or to development of specific methods, and they all read and approved the final manuscript.
